# Pharmacokinetic-pharmacodynamic target attainment with continuous infusion piperacillin in patients admitted to the ICU with hospital-acquired pneumonia

**DOI:** 10.1128/aac.01760-25

**Published:** 2025-12-29

**Authors:** Marta Zurawska, Adrian Valadez, Emma Harlan, Ryan Williamson, Marc H. Scheetz, Michael N. Neely, Paul R. Yarnold, Mengjia Kang, Helen K. Donnelly, Franciso Martinez, Erin Korth, Rachel L. Medernach, Sophia H. Nozick, Alan R. Hauser, Egon A. Ozer, Estefani Diaz, Alexander V. Misharin, Richard G. Wunderink, Nathaniel J. Rhodes

**Affiliations:** 1 Department of Pharmacy Practice, Midwestern University, College of Pharmacy 15475, Downers Grove, Illinois, USA; 2 Pharmacometrics Center of Excellence, Midwestern University 69281, Downers Grove, Illinois, USA; 3 Department of Pharmacy, Northwestern Memorial Hospital, Chicago, Illinois, USA; 4 Departments of Pharmacology and Biomedical Sciences, College of Graduate Studies, Midwestern University 69281, Downers Grove, Illinois, USA; 5 Keck School of Medicine, University of Southern California 5116, Los Angeles, California, USA; 6 Laboratory of Applied Pharmacokinetics and Bioinformatics, The Saban Research Institute, Children's Hospital of Los Angeles 466592, Los Angeles, California, USA; 7 Optimal Data Analysis, LLC, Chicago, Illinois, USA; 8 Division of Pulmonary and Critical Care Medicine, Department of Medicine, Northwestern University, Feinberg School of Medicine 166943, Chicago, Illinois, USA; 9 Division of Infectious Diseases, Department of Internal Medicine, Rush University Medical Center 2468, Chicago, Illinois, USA; 10 Department of Microbiology-Immunology, Northwestern University, Feinberg School of Medicine 547641, Chicago, Illinois, USA; 11 Division of Infectious Diseases, Department of Medicine, Northwestern University, Feinberg School of Medicine 166943, Chicago, Illinois, USA; 12 Center for Pathogen Genomics and Microbial Evolution, Havey Institute for Global Health, Feinberg School of Medicine, Northwestern University 12244, Chicago, Illinois, USA; 13 Robert H. Lurie Comprehensive Cancer Research Center, Feinberg School of Medicine, Northwestern University 12244, Chicago, Illinois, USA; Providence Portland Medical Center, Portland, Oregon, USA

**Keywords:** population pharmacokinetics, piperacillin, target attainment, renal dysfunction

## Abstract

Optimizing β-lactam antibiotic exposure in critically ill patients with hospital-acquired pneumonia (HAP) remains a challenge due to significant pharmacokinetic variability, particularly in the setting of renal dysfunction and replacement therapies. Continuous infusion (CI) of piperacillin/tazobactam aims to improve pharmacodynamic target attainment, though both subtherapeutic and potentially toxic concentrations have been reported in practice. We developed a population pharmacokinetic model of piperacillin using 162 plasma samples from 35 intensive care unit (ICU) patients with HAP, including those receiving continuous renal replacement therapy (CRRT). Piperacillin concentrations were quantified using a validated LC-MS method. A one-compartment model parameterized with renal and non-renal clearance was implemented in Monolix, incorporating creatinine clearance (CrCL), CRRT effluent flow rate, and intermittent hemodialysis as key covariates. Monte Carlo simulations in Simulx evaluated steady-state drug exposures following renal dose-adjusted CI regimens. Simulation showed that renally adjusted lower doses administered via CI (3–9 g/day) achieved target concentrations in 74–82% of patients with CrCL ≤75 mL/min. Higher doses (6–12 g/day) resulted in >20% of patients exceeding 96 mg/L across all renal strata. Among CRRT patients, lower doses provided a 100% probability of maintaining targeted piperacillin concentrations. In patients with supra-normal renal function (i.e., CrCL = 150 mL/min), low-dose CI regimens yielded a 6.1% probability of underexposure, compared to 2.7% with high-dose. CI PIP dosing based on CrCL results in variable exposures among ICU patients. Individualized dosing of PIP may be required to optimize efficacy and minimize toxicity in ICU patients treated with CI dosing.

## INTRODUCTION

Infections in critically ill patients, such as those with hospital-acquired pneumonia (HAP), require early initiation of broad-spectrum antibiotics to improve morbidity and mortality (1). β-lactam antibiotics are widely used as first-line antibiotics in this patient population. These antimicrobials exhibit a time-dependent pharmacokinetic/pharmacodynamic (PK/PD) profile, yielding optimized bacterial killing when free drug concentrations remain above the minimum inhibitory concentration (MIC) of the infecting pathogen (*f*T_>MIC_) (2). The proposed PK/PD target for beta-lactams is 40–70% of the dosing interval based on pre-clinical models (3, 4). Limited clinical data suggest that β-lactam concentrations in plasma should ideally remain above the target pathogen MIC for at least 50% of the dosing interval with greater benefits observed when 100% *f*T_>MIC_ is attained in plasma (5). For critically ill patients, guidelines suggest targeting four times the MIC (100% *f*T_>4× MIC_) for empiric treatment (6). Clinical trials report that continuous infusion (CI) of β-lactam antibiotics is more likely to yield reduced mortality and greater clinical cure compared to conventional intermittent dosing attributable to optimizing PK/PD exposures in patients with sepsis and septic shock (7, 8). However, even when CI dosing is used, individual patients can experience significant variability in exposures, leading to plasma concentrations ranging from subtherapeutic to potentially toxic (9–11).

Piperacillin (PIP), combined with the β-lactamase inhibitor tazobactam, is among the most common broad-spectrum β-lactams used in patients with nosocomial infections including pneumonia (12–14). While extended infusion dosing of PIP combined with tazobactam over 3 or 4 h has become the norm in many hospitals, CI of PIP can optimize PK/PD. Dulhunty et al. reported that CIs of PIP in patients with septic shock resulted in numerically, though not significantly, lower odds of death (OR: 0.89, 95% CI: 0.79–1.01) (7). Prolonged infusions (i.e., continuous or extended interval dosing) of PIP had a comparable effect (OR: 0.86, 95% CI: 0.58–1.10) in a systematic review and meta-analysis (8). In a prospective randomized controlled trial, Hagel et al. found that individualized dosing of PIP using therapeutic drug monitoring (TDM) paired with CI dosing resulted in numerically lower mortality rates overall and improved odds of clinical cure (10). Post hoc analysis revealed significantly increased risk of death (OR: 4.21, 95% CI: 1.4–12.5) among patients with PIP concentrations exceeding 96 mg/L. Thus, both subtherapeutic concentrations and overdosing can impact clinical outcomes (9). As critically ill patients have dynamic renal states and frequently require continuous renal replacement therapy (CRRT), it is not surprising that intensive care unit (ICU) patients can experience drug accumulation and variability in target concentrations (15).

In this study, we developed a population PK model of PIP in critically ill patients with HAP and applied the model to CI dosing strategies. The probability of subtherapeutic and supratherapeutic exposures across a range of renal states and CRRT modalities was evaluated from simulations to identify patient subpopulations at risk for over- and underdosing of PIP.

## MATERIALS AND METHODS

### Patients

Critically ill patients with HAP admitted to the medical ICU at Northwestern Memorial Hospital and who received piperacillin-tazobactam (TZP) were enrolled between June 2018 and May 2023. Indication-based antimicrobial dose selection and renal dose adjustments were made according to institutional protocols at the discretion of the primary treatment team (asp.nm.org).

In this study, residual blood samples left over from clinical care were opportunistically captured from patients enrolled in the Successful Clinical Response in Pneumonia Therapy (SCRIPT, U19AI135964) (https://script.northwestern.edu/database/) and RX-SCRIPT studies, which focused on β-lactam treated patients with and without CRRT (R21AI174159 and R01AI158530, respectively). Collection times were recorded from the electronic medical record for residual blood samples. Patients requiring concurrent extracorporeal membrane oxygenation (ECMO) were excluded from the present study. All patients or their legally authorized representative provided written informed consent prior to being enrolled in SCRIPT and RX-SCRIPT. The protocol was approved by the Northwestern University Institutional Review Board (STU002048680). Plasma samples were processed according to protocol and stored for batch PK analysis at −80°C (16).

### Bioanalysis of drug concentrations

Total PIP was quantified using Agilent 1260 Infinity II liquid chromatography system coupled with Agilent Ultivo Triple Quadrupole mass spectrometer (LC-MS). The analytical separation was achieved using an Agilent Infinity Lab Poroshell 120 EC-C18 column (100 mm ×  3.0 mm  ×  2.7 μm). Mobile phase A was 0.1% formic acid in water, and mobile phase B was 100% LC/MS-grade acetonitrile. Transitions (*m*/*z*) for PIP were: quantitation 518.2 → 143, qualification 518.2 → 160.0; PIP-d^5^ (*m*/*z*: 523.3 → 148.1 and 160) served as an internal standard. The assay was linear from 0.5 to 80 mg/L; samples that were above the limit of quantitation (ALQ) were diluted to fall within the linear range. Accuracy (inter-day: 97.1–104%; intra-day: 89.3–119%) and precision (inter-day coefficient of variation [CV%]: 2.17–15.2%; intra-day CV%: 1.01–6.4%) met Food and Drug Administration requirements for bioanalytical method validation (17). PIP concentrations below the lower limit of quantitation (LLOQ) of 0.5 mg/L were considered below the limit of quantitation (BLQ) and modeled in Monolix using interval censoring, with the likelihood estimated over the interval [0, LLOQ]. Analysis of tazobactam in plasma is described by Williamson et al. (18).

### Population PK modeling

Population PK modeling of PIP was performed using Monolix 2024R1. The system was described using a series of differential equations. Base one-compartment and two-compartment models were evaluated, with first-order elimination from the central compartment. Given the opportunistic nature of blood sampling (typically collected in the morning) relative to hemodialysis (HD) times (typically conducted in the late afternoon), HD clearance was fixed to literature values (19). The tazobactam population PK model is described by Williamson et al. (18).

### Covariate, regressor, and error models

Due to the opportunistic nature of PK sampling over a long time horizon that did not strictly align to specific occasions across subjects, we chose to use the regressor approach available in Monolix to model time-varying covariates. Planned regressors evaluated included total body weight on Vd, CrCL on CL, and effluent flow rate on clearance in CRRT. Planned covariates evaluated included age, body surface area (BSA), and sex. Multiple clearance pathways, non-CRRT, CRRT, and HD clearances were evaluated *a priori*. In the base model, total clearance was described by the following piece-wise equations:


(1) $$CL_{total}=CL_{nonCRRT}+\;CL_{CRRT}+\;CL_{HD}$$

(2) $$CL_{nonCRRT}=CL_{renal}+\;CL_{nonrenal}$$

Covariate models considered continuous variables (e.g., age, weight, and BSA) using power models, for example:


(3) $$CL_{individual\left(i\right)}=\;CL_{population}\times {\left(\frac{Covariate_{i}}{Referent}\right)}^{beta\_covariate}\times e^{\eta CL,i}$$

where ηCL,i was the random effect estimate for clearance.

Fractional changes from the referent category were evaluated using an indicator variable for categorical variables (e.g., sex), for example:


(4) $$V_{individual\left(i\right)}=\;V_{population}\times \left(1-Covariate_{i}\times beta\_covariate\right)\times e^{\eta V,i}$$

where ηV,i was the random effect estimate for volume.

Covariates and regressors were evaluated sequentially with a change in the objective function value of 3.84 required for inclusion and 6.64 for backward elimination. Regressors were considered time-varying covariates (e.g., CrCL, CRRT, and HD) and were integrated into the base model. Final model selection was based on the minimization of the BICc, model diagnostics including condition number and the rule of parsimony. Time after dose visual predictive checks (VPCs) (20) were generated in R using observed and simulated (*n* = 500 replicates) PIP concentration-time profiles (six equal-sized bins were used). Observed concentrations that were BLQ (i.e., censored) were excluded from percentile calculations. Uncertainty in the final model parameters was quantified using non-parametric (*n* = 1,000) bootstrap resamples.

### Simulations

The final covariate-adjusted population model was translated into Simulx 2024R1. Monte Carlo simulations were conducted to assess the PK/PD target attainment 48 h into a renal-adjusted CI dosing regimen after administration of a 4 g loading dose (LD). Renal function was categorized based on the Cockcroft-Gault calculated creatinine clearance (CrCL) (21), with dose adjustments made at cutoff values of 25, 50, 75, and 150 mL/min. For patients receiving CRRT, effluent rates of 25 and 35 L/kg/h were simulated, assuming a measured total body weight of 70 kg. We also evaluated total effluent flow rates based on larger body weights (e.g., 91 and 126 kg) in a sensitivity analysis. For each dose regimen and renal disposition, 1,000 patient simulations were completed using the final model population parameters and corresponding uncertainties. Continuous IV infusion (CI) regimens were evaluated as low dose (3–9 g/day) or high dose (6–12g/day) based on renal disposition (e.g., at a CrCL of 25 mL/min, low-dose and high-dose regimens were 3 and 6 g/day, respectively). The probability of achieving PIP plasma concentrations within predefined therapeutic ranges at 48 h into the infusion was evaluated as follows: desirable: 32–64 mg/L, acceptable: 16–96 mg/L, and excessive: >96 mg/L based on the post hoc analysis revealing increased mortality in the TARGET Trial (10). A sensitivity analysis evaluating PIP concentrations >160 mg/L (22) was also conducted. Simulation results were visualized using the *ggplot2* package for R (version 4.4.2).

## RESULTS

A total of 162 plasma samples were available from 35 patients (Table 1). Of these samples, seven were below the limit of quantitation (BLQ) and were thus handled as censored in the analysis. A total of 16 patients required RRT during PIP treatment, of which 15 required CRRT and one required HD. Among patients requiring CRRT, the highest mean ± SD effluent flow rate was 2.75 ± 1.1 L/h (32 ± 7.8 mL/kg/h). Among patients not requiring RRT, the mean ± SD CrCL was 78 ± 68 mL/min (range: 9–229 mL/min).

**TABLE 1:** Demographics of ICU patients contributing plasma PK samples^*a*^

Demographic (*N* = 35)	Estimate^*b*^
Age (years)	62 ± 16
Total body weight (kg)	79.6 ± 24.1
Body surface area (m^2^)	1.9 ± 0.3
Initial SCr (mg/dL)^*c*^	1.7 ± 1.1
Initial CrCL (mL/min)^*c*^	77.7 ± 68.4
Required an RRT, *n* (%)	16 (46%)
Required HD only, *n* (%)	1 (3%)
Required CRRT, *n* (%)	15 (43%)
Male, *n* (%)	17 (49%)
Female, *n* (%)	18 (51%)

^*a*^ CrCL, Cockcroft-Gault calculated creatinine clearance; CRRT, continuous renal replacement therapy; HD, hemodialysis; RRT, renal replacement therapy; SCr, serum creatinine.

^*b*^ All estimates are mean ± SD unless otherwise indicated.

^*c*^ Non-RRT patients.

### Base model development and model selection

Model development and covariate evaluations are summarized in Table S1. A two-compartment structural model (i.e., Run 3, see Table S1) was evaluated and yielded some improvement in objective function value, but was ultimately rejected owing to the sparse nature of opportunistic plasma sampling which led to unstable estimates of intercompartmental clearance and peripheral volume. Covariate assessment thus proceeded using a one-compartment model structure. Addition of renal (CL_R_) and non-renal (CL_NR_) clearance pathways (Run 4) improved model fit (ΔOFV −24.0); however, high relative standard errors and CV% for inter-individual variability (IIV) were observed with CL_NR_. Removal of IIV on CL_NR_ (Run 5) retained OFV improvements (−25.1) yielding an acceptable condition number. Run 5 was retained as the final model.

### Covariate model

Clearance was modeled as a function of renal function (measured via CrCL), dialysis status (intermittent HD), and CRRT with varying effluent flow rates. The final covariate-adjusted population PK model equations were:


(5) $$CL_{nonCRRT_{ind}}=\left(CL_{CRCL_{pop}}\times \left(\frac{CRCL}{120mL/min}\right)+CL_{NR_{pop}}\right)\times \left(1-CRRT\right)\times \left(1-HD\right)$$

(6) $$CL_{CRRT_{ind}}=CL_{CRRT_{pop}}\times \left(\frac{FLOW}{2L/h}\right)\times CRRT$$

(7) $$CL_{HD_{ind}}=CL_{HD_{pop}}\times HD$$

In these equations, HD and CRRT are indicator variables (either on or off), and FLOW was the CRRT effluent flow rate in mL/h. Intra-HD clearance for PIP was taken from the literature and fixed at 4.16 L/h (19).

### Final population PK model parameters

**Fig 1:**
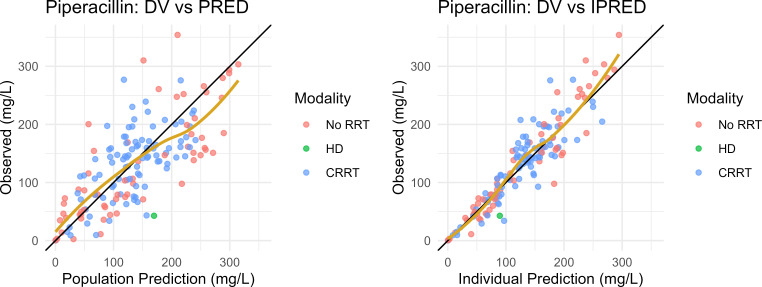
Goodness-of-fit plots for the final population pharmacokinetic model of piperacillin. Observed plasma concentrations of piperacillin (DV) on the *y*-axis to model-predicted concentrations on the *x*-axis. Left: Population PRED. Right: Empirical Bayes Estimates (iPRED). Goldenrod line: loess spline.

**Fig 2:**
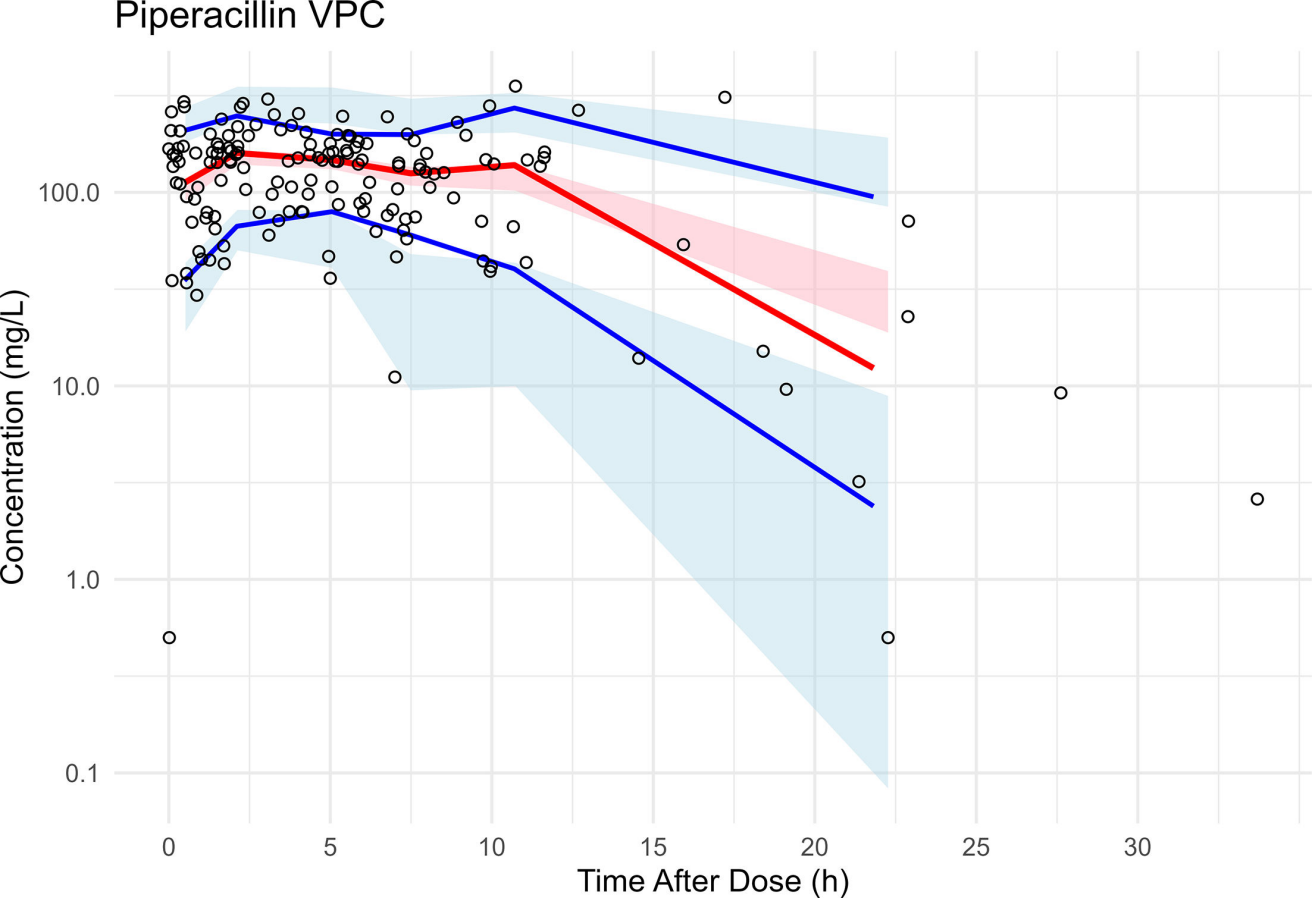
Visual predictive check (VPC) for the final PIP population PK model. Prediction-corrected VPC. Shaded ribbons show the prediction-corrected simulated concentrations from the final model across 500 replicates: blue = 10th–90th percentile, pink = interquartile range. Solid lines show the median (red) and 10th/90th percentiles (blue) of the observed, prediction-corrected concentrations. Circles: observations.

Final PK model parameter estimates, and their 95% bootstrap CIs, are presented in Table 2. The population typical estimates for PIP Vd, CL_R_, and CL_NR_ were 28.1, 5.77, and 0.65 L/h, respectively. For patients on CRRT, the population typical estimates for PIP CL were 3.41 L/h based on a 2 L/h effluent flow rate. IIV was well estimated (RSE < 30%) with CV% for Vd, CL_R_, and CL_CRRT_ of 33%, 70%, and 25%, respectively (Table 2). Shrinkage ranged from low (21% CL_renal_) to moderate (44% CL_CRRT_) for clearance estimates and was moderate for Vd (39%). Goodness-of-fit plots for the population and Bayesian posterior predictions of the PIP model are given in Fig. 1. The model yielded acceptable predictive performance in pc-VPC analysis (Fig. 2). TAZ model parameter estimates are described by Williamson et al. (18).

**TABLE 2:** Population model parameters and estimates for piperacillin^*a*^^,^^*b*^

Parameter	Estimate, mean	Non-parametric bootstrap resampling (*n* = 1,000), median	RSE (%)	Non-parametric bootstrap resampling (*n* = 1,000)	
				Lower 2.5th CI	Upper 97.5th CI
**Fixed effects**					
Vd, L	28.1	28.56	13.0%	23.7	37.3
CL_CrCL_, L/h (reference, 120 mL/min)	5.1	5.3	23.1%	3.0	8.0
Non-renal CL, L/h	0.65	0.74	36.2%	0.33	1.35
CL_HD_, L/h	4.2	Fixed	Fixed	Fixed	Fixed
CL_CRRT_, L/h	3.4	3.45	8.9%	2.9	4.1
**Interindividual variability**					
**Random effects**	**C.V. %**				
*ω*_Vd_	32.9%	32.9%	32.3%	14.1%	58.2%
*ω*_CLCrCL_	69.6%	70.1%	27.6%	42.8%	163%
*ω*_CLCRRT_	24.5%	24.6%	33.9%	9.4%	41.7%
Residual error					
Proportional error (%)	25%	24%	10.2%	19.2%	29.6%

^*a*^ Population mean PK parameters and associated uncertainties for piperacillin. Fixed effects reported as mean estimate; interindividual variation (*ω*) reported as CV %. Piperacillin clearance was estimated as a time-varying function with population PK parameters defined for HD (fixed), renal (CrCL), non-renal, and CRRT effects. Upper 97.5th and lower 2.5th percentiles from bootstrap replicates.

^*b*^ CL, clearance; CrCL, Cockcroft Gault estimated creatinine clearance; CRRT, continuous renal replacement; HD, hemodialysis; Vd: volume of distribution.

### Simulations

**Fig 3:**
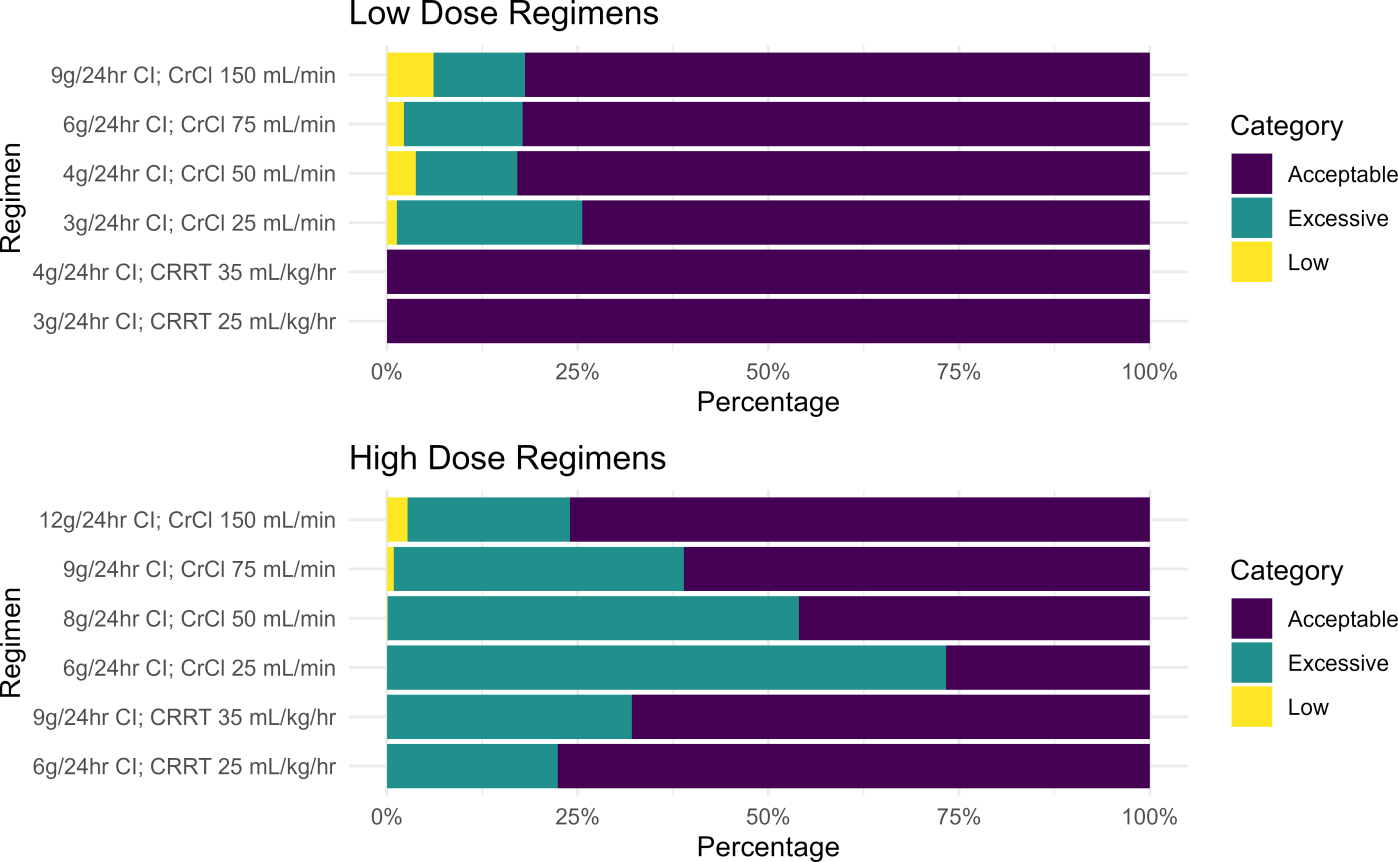
Probability of simulated steady-state piperacillin concentrations falling below, within, or above a target range of 16–96 mg/L. Piperacillin concentrations were categorized as low (<16 mg/L), acceptable (16–96 mg/L), or excessive (>96 mg/L) based on the post hoc analysis of the TARGET Trial.

Simulation revealed that after receipt of an LD and 48 h of CI treatment, patients were more likely to have PIP concentrations in the target range (16–96 mg/L) with a lower-dose regimen versus a higher-dose regimen (Fig. 3). Among patients with high to preserved renal function (CrCL = 150 mL/min), the probability of having a steady state PIP concentration below 16 mg/L increased marginally (6.1% vs 2.7%). Lower-dose regimens had a <5% probability of falling below 16 mg/L for all other renal states (Fig. 3). In contradistinction, higher-dose regimens had probabilities of exceeding 96 mg/L ranging from 21.3% to 73.3%, whereas lower-dose regimens showed probabilities ranging from 0% to 24.3%.

Lower-dose regimens performed best for patients on CRRT using fixed body weights of 70 kg to define effluent rates of 25 and 35 mL/kg/h. Among larger patients (e.g., total body weights of 91 and 126 kg), adequate target attainment was achieved with 9 and 12 g/day at effluent flow rates of 35 mL/kg/h and performed similarly to 6 g/day for a 70 kg patient at the same weight-based effluent flow rate (Fig. S1). For patients with CrCL of 25 mL/min, the probability of exceeding 96 mg/L was >20% despite application of a lower 24-h dose (Fig. 3), suggesting a possible role for therapeutic monitoring of PIP within this renal strata.

We also evaluated the probability that concentration would exceed 160 mg/L in a sensitivity analysis of toxicity potential as shown in Table S2. The probability of exceeding 160 mg/L for patients receiving high-dose CI regimens was greatest with CRCL of 25 mL/min (31.9%), followed by CRCL of 50 mL/min (20.4%) and 75 mL/min (10.1%). For all other doses and renal dispositions evaluated, the probability of exceeding 160 mL/min was <6%.

## DISCUSSION

We observed a high degree of variability in PIP PK among TZP-treated patients owing largely to inter-individual variations in renal clearance for individuals not requiring CRRT. In contrast, CRRT clearance was less variable between patients (i.e., *ω*_CLCRRT_ CV = 24.5%), and was largely explained by differences in effluent flow rate. Notably, CRRT clearance in the setting of continuous venous hemodiafiltration is both diffusive and convective (23), and recent studies report that effluent flow rates in practice range from less than 20 to over 30 mL/kg/h (mean ± SD: 26±7.4 mL/kg/h) (24). Thus, total effluent flow rates will account for differences in patient body weight. We varied body weights in our simulations to better capture real-world exposures at various effluent flow rates. Our population mean CRRT clearance estimate was 3.4 L/h standardized to a 2 L/hr effluent flow rate, which is similar to previous studies when residual renal function is minimal (25–27). Whereas effluent rates modified CRRT clearance in our sample, we did not observe a significant effect of body weight on Vd. Renal clearance and non-renal clearance terms were estimated for PIP yielding significant improvements in model fits (Table S1). This separation of clearance into renal and non-renal pathways is consistent with prior studies (28–30). Unlike the study by Felton et al. (31), we were unable to accurately estimate non-linear Michaelis-Menten (MM) or parallel first-order MM clearance terms (data not shown), likely attributable to limitations of available PK sampling.

We simulated a range of CI dosing strategies revealing highly variable PK exposures among patients not requiring CRRT. Our findings indicate that high-dose CI regimens are likely to be excessive for over 25% of patients with mild renal impairment (CrCL ≤ 75 mL/min), suggesting the need for TDM to achieve PIP dose individualization in the ICU setting. The importance of controlling individual patient exposures with high-dose CI PIP is underscored by post hoc findings of increased mortality with supratherapeutic PIP concentrations in the TARGET trial, suggesting a possible relationship between elevated concentrations and poor outcomes (10). A systematic review by Mohd Rozi et al. reported increased mortality among CRRT patients treated with PIP experiencing excessive concentrations (e.g., >100 mg/L) (32). Roberts et al. found renal replacement therapy to be an independent risk factor for mortality and lack of clinical cure among patients enrolled in trials comparing continuous and intermittent infusion dosing (33). Additional studies are needed to clarify whether and to what extent these relationships are causal versus associative, and what impact renal replacement has on site of infection PK/PD attainment. Nevertheless, our simulations projected a wide range of exposures from both high and low dose CI regimens, revealing a need for optimized dosing strategies to avoid excessive (>96 mg/L) concentrations.

Variability in PIP concentrations among critically ill patients has been observed previously among patients in the ICU. Zander et al. reported that in an ICU cohort including CRRT and ECMO patients, ≥123-fold IIV existed for PIP TDM trough samples taken across four distinct study days, and trough values on day 1 of therapy were sub-optimal in up to 47% of consecutively enrolled ICU patients (34). Of note, our model estimates suggest that CRRT-related clearance is relatively less variable between CRRT patients compared to variability between patients with similar estimated (21) renal clearance. Roger et al. found that PIP CRRT-related clearance had an IQR of 2.7–3.2 L/h yielding 100% *f*T_>MIC_ in their cohort (27). Reeder et al. identified a population PK model of PIP and reported a bootstrap 95% CI for CRRT CL to be 2.65–4.43 L/h (30), overlapping our model’s bootstrap 95% CI for CRRT CL (2.9–4.1 L/h) (Table 2). Thus, CRRT-related clearance appears to be more consistent and may result in excessive concentrations when doses are increased out of proportion to extracorporeal drug clearance. This hypothesis is supported by data from Economou et al., who found that among CRRT patients undergoing PIP TDM, those receiving higher doses (4.5 g IV q6 h) required dose decreases 67% of the time compared to those receiving every q8 h to q12 h dosing (22% and 14% dose reductions, respectively) (15).

Strengths of our study included the integration of longitudinal PK data using nonlinear mixed-effects modeling and the diversity of our patient cohort, which together allowed evaluation of a wide range of renal states and extracorporeal clearance. Limitations were that dosing for patients in our cohort was based on estimates of patient renal function, TDM was not incorporated into clinical decision-making, and real-time concentration data were not available to guide dose adjustments. While the study cohort was relatively small (*n* = 35), it included a diverse set of patients with varying degrees of renal function and CRRT exposure and was adequate for estimating renal, non-renal, and CRRT CL with adequate precision and statistical power. The opportunistic nature of PK sampling in this study was adequate to characterize PIP during the infusion period and early elimination phase as shown in the time after dose VPC (Fig. 2). Forthcoming research will evaluate our model by external validation. Ideally, estimates of CRRT-specific clearance for PIP would be derived from plasma and effluent concentrations. Additional ongoing studies will clarify the impact of CRRT effluent flow rates on PIP clearance and will serve to refine our current estimates. We fixed HD clearance to literature-based estimates due to sparse sampling around dialysis sessions, but this approach appeared reasonable given the homogeneity of intra-dialytic clearance. Planned future research will evaluate the impact of patient exposures and pathogen MICs on clinical outcomes, and the impact of applying low-dose CI regimens on joint TZP PK/PD attainment.

### Conclusion

We identified a population PK model for PIP and simulated high-dose and low-dose CI regimens in an ICU population. Greater than 25% of simulated PIP-treated patients with a CrCL of 25 mL/min were at risk of excessive (>96 mg/L) concentrations despite receiving a low-dose regimen, whereas patients with supranormal renal function (CrCL = 150 mL/min) were at risk of having inadequate (<16 mg/L) concentrations. The findings suggest a potential role of TDM in these subpopulations, and models such as the one described herein may be useful in optimizing dosing.

## SUPPLEMENTAL MATERIAL

Data S1 aac.01760-25-s0001.xlsx Full list of SCRIPT Investigators acknowledged.

Supplemental material aac.01760-25-s0002.pdf Tables S1 and S2, Fig. S1 to S3.

ASM does not own the copyrights to Supplemental Material that may be linked to, or accessed through, an article. The authors have granted ASM a non-exclusive, world-wide license to publish the Supplemental Material files. Please contact the corresponding author directly for reuse.
