# Supplementary material for: Pharmacokinetic-pharmacodynamic target attainment with continuous infusion piperacillin in patients admitted to the ICU with hospital-acquired pneumonia
Source: Antimicrob Agents Chemother. 2025 Dec 29;70(2):e01760-25. doi: 10.1128/aac.01760-25 (PMC12888853; doi:10.1128/aac.01760-25)
Supplement: Supplemental material — Tables S1 and S2, Fig. S1 to S3. [file aac.01760-25-s0002.pdf]

Supplemental Figures:

Figure S1. Individual weighted residuals of piperacillin in plasma

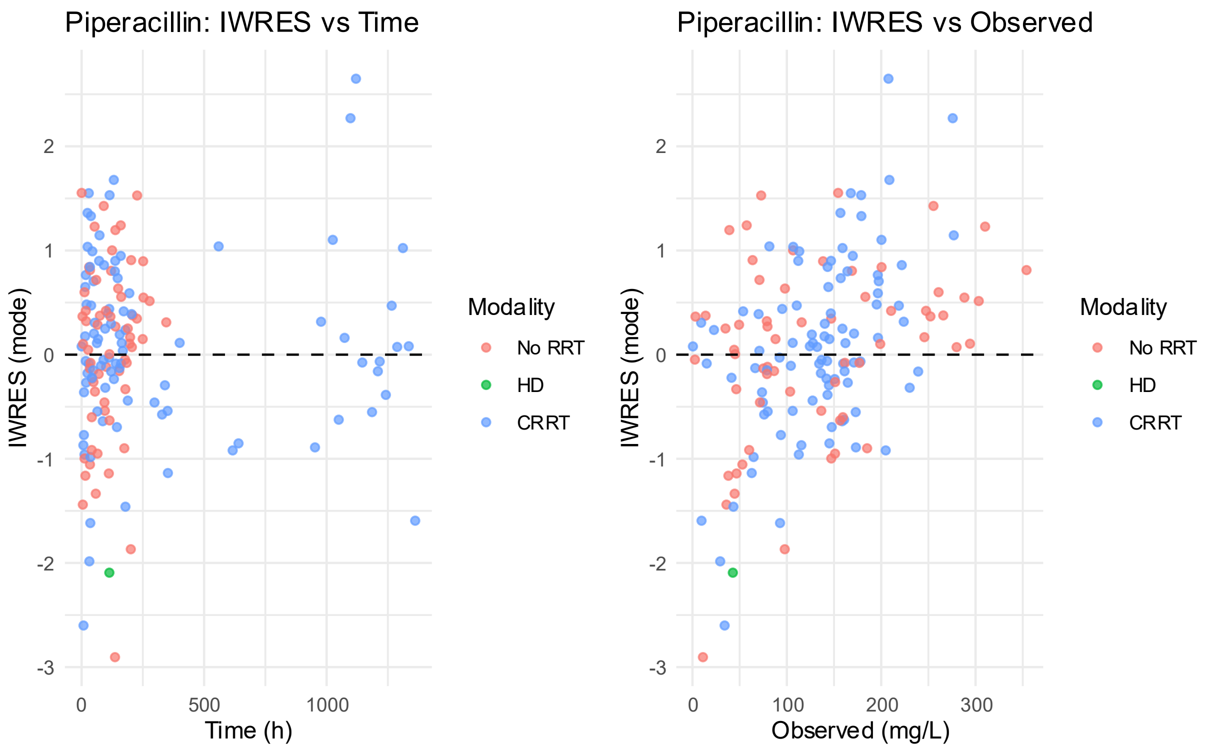

Figure legend: Observed time of sample collection vs residual (left) and observed plasma concentrations of piperacillin (DV) vs residual (right). Modal Empirical Bayes Estimates were used to generate individual weighted residuals (IWRES).

Figure S2. Representative simulated piperacillin plasma concentrations over the first 48 hours with high and low dose CI dosing regimens.

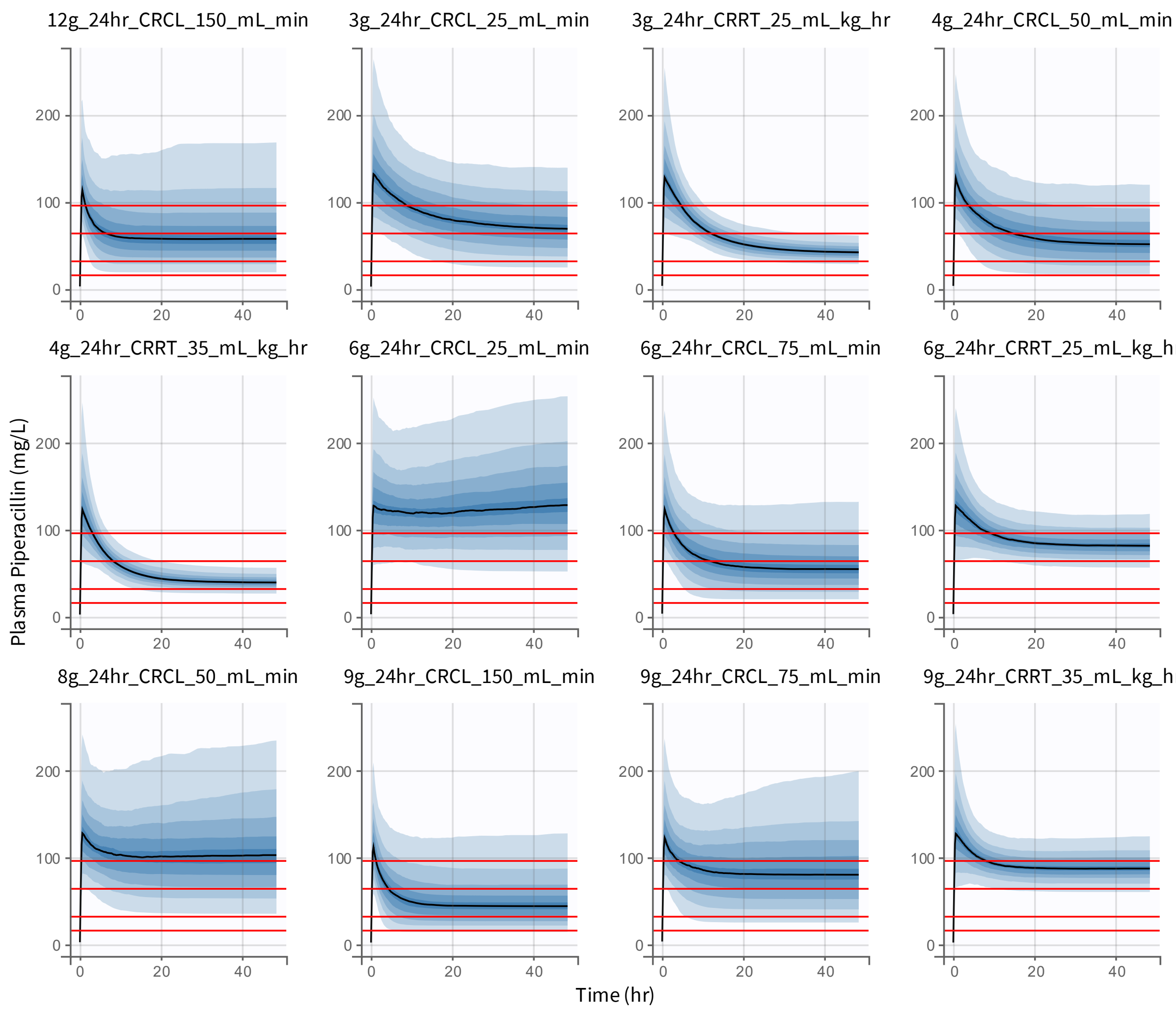

Figure legend: Simulated piperacillin (PIP) concentration distributions were generated. PIP concentrations at 48 hr were categorized as low (<16 mg/L), acceptable (16–96 mg/L), or excessive (>96 mg/L) based on the post-hoc analysis of TARGET. Visual cues (red lines) added to distinguish PIP concentrations of 16, 32, 64, and 96 mg/L.

Figure S3. Probability of simulated steady-state piperacillin concentrations falling below, within, or above a target range of 16 to 96 mg/L for patients on CRRT at 35mL/kg/hr.

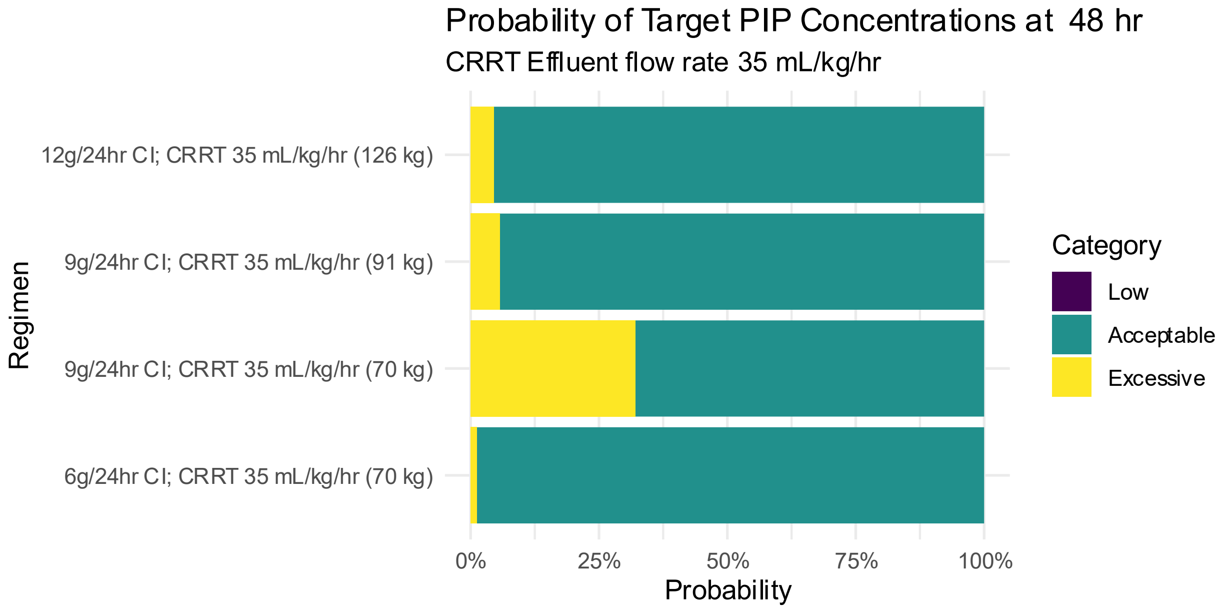

Figure legend: Piperacillin concentrations were categorized as low (<16 mg/L), acceptable (16–96 mg/L), or excessive (>96 mg/L) based on the post-hoc analysis of the TARGET Trial.

Table S1. Piperacillin-tazobactam structural and covariate model development build

Run	Model	-2*LL (IS)	BICc (IS)	Forward Δ OFV	Forward Referent	Backward Δ OFV	Backward Referent	Selection	Observation model	Comments
**1**	**1 compartment base**	2436.76	2515.52	NA	NA				**y1**comb1 **y2**comb1	Base model 1 comp, regressor, high RSE on additive pip
**2**	**Run 1 with updated error models**	2436.01	2509.03	-0.75	1			Basis	**y1**prop **y2**comb1	Base model 1 comp based on run 1. Prop model pip, comb 1 for taz
**3**	**2 compartment base**	2399.58	2495.57	-36.43	2	11.37	5	Reject	**y1**prop **y2**comb1	Base 2 comp model based on run 2. Unable to estimate peripheral V.
**4**	**Run 2 with CLR and CLNR**	2412.05	2503.67	-23.96	2	-1.1	5	Accept	**y1**prop **y2**comb1	Base model 1 comp based on run 2 adding non-renal CL. High RSE on IIV and CLNR pop est.
**5**	**Run 4 with no IIV on CLNR**	2410.95	2495.46	-25.06	2	NA	NA	Final	**y1**prop **y2**comb1	Base model 1 comp based on run 2 adding non-renal CL no IIV. Condition number 67
**6**	**Run 5 with combined error**	2412.14	2502.39	1.19	5				**y1**comb1 **y2**comb1	Base model 1 comp based on run 4 testing comb1 error all. BICC inc.
**7**	**Run 5 with proportional error**	2429.58	2508.34	18.63	5				**y1**prop **y2**prop	Base model 1 comp based on run 4 testing prop error all, inc BICC.
**8**	**Run 5 with WT on V regressor**	2416.22	2500.73	5.27	5				**y1**prop **y2**comb1	Base model 1 comp based on run 3 scaling V to WT/70. Condition number 43.9 BICC increased.
**9**	**Run 5 with allometric scaling**	2415.18	2499.69	4.23	5				**y1**prop **y2**comb1	Base model 1 comp based on run 4 allometric WT/70. inc BICC and high RSE on all parameters.
**10**	**Run 5 with CRRT on Vd piecewise**	2411.76	2514.86	0.81	5				**y1**prop **y2**comb1	Base model 1 comp based on run 4 adding piecewise V for CRRT. High RSE on Vs inc BICC.
**11**	**Run 5 with CRRT on Vd fractional change**	2411.3	2507.29	0.35	5				**y1**prop **y2**comb1	Base model 1 comp based on run 4 adding linearlized V on CRRT. High RSE on V inc BICC

Table legend: Sequence of structural and error model development runs, including objective function values, model selection criteria, and key observations. For each run, the model type, changes implemented, and evaluation statistics are listed. Forward and backward inclusion steps are indicated along with the corresponding change in objective function value (ΔOFV) and referent model. The final selected model is noted.

Abbreviations: ΔOFV, change in objective function value; BICc (IS), corrected Bayesian Information Criterion by Importance Sampling; CLR, renal clearance; CLNR, non-renal clearance; IIV, inter-individual variability; Vd, volume of distribution; WT, body weight; CRRT, continuous renal replacement therapy; comb1, combined error model (additive + proportional error); prop; proportional error model; RSE, relative standard error; NA, not applicable.

Table S2. Probability of exceeding piperacillin concentrations of 160 mg/L with CI dosing

Probability exceeding 160 µg/mL at 48 hr

**Renal state**	**Probability Cpip > 160 with High Dose CI**	**Probability Cpip > 160 with Low Dose CI**
CRRT 25 mL/kg/hr	0.0	0.0
CRRT 35 mL/kg/hr	0.0	0.0
CrCl 25 mL/min	31.9	1.3
CrCl 50 mL/min	20.4	0.7
CrCl 75 mL/min	10.1	2.0
CrCl 150 mL/min	5.9	2.3
